# Positive Youth Development in Croatia: School and Family Factors Associated With Mental Health of Croatian Adolescents

**DOI:** 10.3389/fpsyg.2020.611169

**Published:** 2021-01-15

**Authors:** Miranda Novak, Nicholas J. Parr, Martina Ferić, Josipa Mihić, Valentina Kranželić

**Affiliations:** ^1^Laboratory for Prevention Research, Department for Behavioral Disorders, Faculty of Education and Rehabilitation Sciences, University of Zagreb, Zagreb, Croatia; ^2^Prevention Science Institute, University of Oregon, Eugene, OR, United States

**Keywords:** positive youth development, internalizing problems, adolescence, school, family, youth mental health

## Abstract

**Introduction:**

A framework for understanding the interrelationship of individual and environmental factors that influence adolescent health and well-being, as well as opportunities for policy-level interventions, is known as Positive Youth Development (PYD). The current study represents one of the largest studies of Croatian adolescents to date, and aimed to examine associations between school and family factors linked to PYD, and mental health outcomes experienced by Croatian youth.

**Methods:**

A multi-site survey study was conducted among adolescents (*N* = 9,655) residing in the five most populous cities in Croatia, with the aim of examining cross-sectional associations of family and school factors with adolescent mental health. The mean age of participants was 16.3 years (*SD* = 1.2), and 52.5% of participants were female. School and family factors included school attachment, school commitment, family communication, and family satisfaction. Depression, anxiety, and stress were assessed as outcomes. Multigroup structural equation modeling (SEM) was used to examine relations of interest among female and male adolescents.

**Results:**

Among school factors, increased school attachment was found to be significantly associated with reduced depression, anxiety, and stress for female adolescents, and with decreased depression and stress for male adolescents. Increased school commitment was significantly associated with decreased depression and anxiety for female adolescents; conversely, an increase in school commitment was associated with an increase in anxiety and stress for male adolescents. Increases in family communication were significantly associated with reduced depression, anxiety, and stress only for male adolescents, while increased family satisfaction was significantly associated with reduced depression, anxiety, and stress for female adolescents and with decreased depression and stress for male adolescents.

**Conclusion:**

Findings suggest that interventions for mental health promotion and prevention of internalizing problems should address both school and family contexts, and may be more effective when accounting for differing developmental experiences of female and male adolescents.

## Introduction

Worldwide, the global burden of mental health conditions among adolescents is increasing. Studies have shown that around half of mental health problems start by middle adolescence while 75% of adult mental health issues start before the age of 24 (Kessler et al., 2007; Jones, 2013). It is estimated that, overall, more than 20% of youth could experience mental health issues (Costello et al., 2003; Merikangas et al., 2010; Jones, 2013; Dooley et al., 2015). The most prevalent mental health problems in adolescence are internalizing difficulties (Merikangas et al., 2010; Achenbach et al., 2016), including anxiety and mood disorders characterized by negative affect, poor mood, low self-esteem, overt behavioral control issues, and other inner-directed symptoms (Achenbach et al., 2016). Among youth aged 13–18, up to 32% have anxiety disorders and 14.3% have mood disorders (Merikangas et al., 2010), and the prevalence of these disorders is increasing, particularly for adolescent females (Bor et al., 2014).

Physiological and psychological maturation during adolescence alters cognitive, emotional, and motivational processes, and these developmental changes interact with other processes that influence adolescents’ mental and behavioral health, including individual traits, family and social environments, and the broader social and economic climate (National Academies of Sciences, Engineering, and Medicine, 2019). A framework for understanding the interrelationship of individual and environmental factors that influence adolescent health and well-being, as well as opportunities for policy-level interventions, is known as Positive Youth Development (PYD; Lerner et al., 2005, 2013; Benson et al., 2006, 2011; Shek et al., 2019; Wiium and Dimitrova, 2019). One of the basic models of PYD is the Five Cs Model (Lerner et al., 2005), which articulates five factors central to healthy development in adolescence: competence, confidence, character, connection, and caring.

Within the Five Cs Model, competence reflects a positive appraisal of personal academic, social, and career abilities that enables success in social tasks, problem-solving, and decision-making (Lerner et al., 2005, 2013). Confidence is seen as a person’s positive belief in their own abilities and includes positive self-image and self-efficacy. Character includes internalization of social rules and norms, sense of right and wrong, and moral integrity. Connection is bidirectional exchange with important individuals in family, school, and community contexts. Caring is a sense of empathy and closeness in one’s social networks.

In the present study, the Five Cs Model is augmented by Benson’s Theory of Developmental Assets (Benson et al., 2006, 2011), which describes internal and external assets relevant to positive adolescent development across gender, environment, race, and ethnicity. These developmental assets lead to healthy outcomes and can be found at both the individual and ecological levels (i.e., in family, school, or community environments). In particular, the current study focuses on the Five Cs Model factor of connection, as well as external assets in school and family environments that facilitate positive relationships and support.

As we will describe, degree of connection to school and family environments, the presence of supportive adults, acceptance, and effective communication, are all factors that relate to adolescent mental health and well-being. The current study sought to improve understanding of the relation between these school and family factors and the mental health of Croatian youth. We examined the association between school external assets (school attachment and school commitment) and family external assets (family communication and family satisfaction) and indicators of adolescent depression, anxiety, and stress. In addition, we examined how these relationships differed by adolescents’ gender.^1^ Based on previous research, we hypothesized that: (1) there would be significant associations between school and family external assets and youth mental health, (2) mental health symptoms would be more severe in adolescent females, and (3) associations between school and family factors and youth mental health indicators would be stronger for adolescent females.

### School Assets

Gomez and Ang (2007) emphasize that schools have the potential to promote PYD because school environments, both academic and non-academic, influence multiple areas of adolescent functioning including identity formation, cognitive and social development, peer relations, and vocational development. Adolescents spend a substantial amount of time in the school environment, increasing the importance of schools as settings for positive interactions and for opportunities that promote PYD (Pittman et al., 2003). Positive school experiences contribute to adolescent resilience and positive development (Olsson et al., 2003). By contrast, prior studies have found adolescent internalizing problems are also associated with school factors (Reddy et al., 2003; Langille et al., 2012; Joyce and Early, 2014; Dooley et al., 2015; Elmelid et al., 2015), including low perceived school connectedness, low teacher support, poor academic achievement and performance, and peer victimization experiences.

School connectedness has been researched under many different terms (Libbey, 2004; Roviš et al., 2016), but despite the differing terminology, school connectedness relates to a psychological state in which students perceive they are supported, respected, and involved in their school environment (Whitlock, 2003). In this study, we operationalize school connectedness as school attachment, or the emotional connection one has with one’s school, teachers, and peers; and school commitment, defined as one’s readiness to invest time and effort in schoolwork (Eitle and Eitle, 2007; Roviš et al., 2016).

As a potential protective factor, strong correlations between school connectedness and PYD have been identified (American School Health Association, 2004). A recent systematic review found evidence that both school connectedness and teacher support predict future emotional health among adolescents (Kidger et al., 2012). In a study of Italian adolescents, Vieno et al. (2004) found that social support from teachers, parents, and peers within the school setting was an important factor in improved student motivation and school satisfaction, which in turn are linked to positive mental well-being outcomes. Students who are less connected to their schools have been shown to have poorer mental health outcomes (Loukas et al., 2009), with one study finding that low school connectedness accounted for 13–18% of emotional distress across various age groups (Resnick et al., 1997).

Many studies have found that a low level of school connectedness is associated with depression and anxiety in youth (Anderman, 2002; Shochet et al., 2006; McGraw et al., 2008; Langille et al., 2012; Dooley et al., 2015). In addition, lower school connectedness has been associated with depression in adolescents in longitudinal studies (Costello et al., 2008; Kidger et al., 2012). Prospective studies have demonstrated that lower levels of school connectedness may be predictive of future depressive symptoms (Kuperminc et al., 2001; Shochet et al., 2006), while Bond et al. (2007) found that a combination of high levels of school and social connectedness was associated with the lowest risk of depression symptoms. An association between school connectedness and suicidal behavior in adolescents has also been identified (Young et al., 2011; Govender et al., 2013). Another dimension of school connectedness, teacher support (i.e., awareness and responsiveness to students’ well-being), has also been associated with depression symptoms (Wang, 2009). Reddy et al. (2003), for example, found that students who reported the greatest declines in teacher–student relationship quality also had the greatest increases in depression. In contrast, students who reported increasing levels of teacher support showed a reduction in depression and growth in self-esteem.

Findings on gender differences in school connectedness are mixed. Some authors report no differences in emotional connection (Langille et al., 2012, 2015; Roviš et al., 2016) while some find female adolescents to be more connected to school (Bond et al., 2007; Elmelid et al., 2015). Shochet et al. (2006) found that school connectedness was associated with depressive symptoms to a greater extent than anxiety symptoms, but found this relation was stronger for female adolescents than for male adolescents. In their study of associations of school connectedness and suicidal behaviors in Canadian adolescents, Langille et al. (2015) found that higher school connectedness was associated with decreased suicidal ideation in both genders but with fewer suicide attempts in females. The authors stressed that the relation between connectedness and mental health outcomes may differ for males and females and recommended that future studies examine those gender differences more closely. Further, several studies have found that female adolescents are perceived as more committed to school than male students, given that males more often have lower grades, higher dropout rates and lower measures of school commitment (Oelsner et al., 2011; Roviš and Bezinović, 2011; Lietaert et al., 2015; Roviš et al., 2016). Lietaert et al. (2015) point to reasons for this perception, including differing types of teacher support as well as lower teacher tolerance of boys’ negative behaviors.

### Family Assets

The family systems perspective states that the family is the most critical system to which an individual belongs (Levin et al., 2012). As a result, the family environment may have a substantial impact on youth development and adolescent mental health (Stormshak et al., 2011; Elgar et al., 2013; Pedersen et al., 2019). Positive parenting practices, in particular, have been found to be associated with fewer depression symptoms (Dooley et al., 2015; Smokowski et al., 2015) and higher emotional well-being, prosocial behavior, and life satisfaction of adolescents (Elgar et al., 2013).

Family satisfaction is a construct that reflects several family assets, and is defined as the degree to which one feels pleased and gratified within one’s family (Olson, 2011). Low family satisfaction is likely to be experienced due to family dysfunction and is prevalent among youth with symptoms of depression (Stavropoulos et al., 2015). Satisfaction with family life has been linked to a variety of aspects of family wellness, including higher family cohesion, adaptability, communication, and overall family functioning (Poff et al., 2010). Family support, harmony, and autonomy have been found to be associated with children’s subjective well-being in several studies (e.g., Paradis et al., 2011; White et al., 2014; Breton et al., 2015; The Children’s Society, 2015, 2017). Further, the results from The Good Childhood Report (The Children’s Society, 2018) showed that youth’s approval of family relationships has the strongest influence (out of five aspects of life) on children’s overall subjective well-being.

Research on adolescents at risk of mental health or conduct problems has identified effective parent–child communication as a protective factor and problematic communication between parents and children as a risk factor for poor adolescent psychosocial adjustment (Xiao et al., 2011). The quality of communication among family members contributes to the quality of the parent–child relationship, which in turn predicts children’s well-being (Broberg, 2012). In a study carried out by Zarnaghash et al. (2013), results showed a significant relationship between adolescent mental health and family communication patterns, including quality of conversation between parents and youth. Other research suggests communication marked by respect among family members reduces the risk of mental health problems (Elgar et al., 2013; Dooley et al., 2015; Smokowski et al., 2015). By contrast, poor family communication is associated with higher adolescent anxiety, depression (Bögels and Brechman-Toussaint, 2006; Davidson and Cardemil, 2009; Smokowski et al., 2015; Withers et al., 2016), and lower self-esteem (Smokowski et al., 2015).

Additionally, there may be important differences in the role of communication based on the gender of adolescents. Levin and Currie (2010), for example, found distinct associations between both mother–child and father–child communication and young people’s life satisfaction. Given this and other research showing that quality of family communication can be a risk or protective factor for male and female adolescents’ mental health and risk behavior, Bireda and Pillay (2018) emphasized the importance of considering the gender of adolescents when examining the relationship between parent–child communication and adolescents’ well-being.

### Context

Croatia is a small European country that has undergone many social and economic changes in recent decades. Some of these changes have precipitated instability in social and family environments. As of 2020, 8.6% of the population was experiencing unemployment (compared to the average unemployment rate of 7.2% in the broader EU^2^), and over 20% of youth aged 15–17 are at risk of poverty (Šućur et al., 2015). Further, one in every three marriages in Croatia ends in divorce (Croatian Bureau of Statistics, 2018).

In contrast to many Western countries that utilize the results of representative national surveys and longitudinal prospective studies to guide interventions for children and adolescents (e.g., Galanti et al., 2016; Landstedt et al., 2016; Christensen et al., 2017), low- and middle-income countries such as Croatia often lack high-quality epidemiological data on adolescent trajectories and PYD. For instance, there are currently no national prevalence estimates of depression, anxiety, and other mental health disorders among adolescents to be compared to European or international indicators.

Compounding the lack of epidemiological estimates, limited research has been conducted to characterize Croatian school and family environments and to understand the relation of school and family factors with youth mental health. Of these, Ajduković et al. (2017) used a nationally representative sample and found a greater number of internalizing problems in female adolescents, adolescents experiencing financial hardship, and those whose parents did not live together. Ajduković et al. (2013) found a high prevalence of parents using physical punishment (72.3%) and high school students reporting physical abuse (40.7% of second year students). Finally, Sušac et al. (2016) report that 35.9% of Croatian children and youth experience peer violence.

Further, to our knowledge, findings on gender-specific associations of school and family factors and mental health outcomes are limited internationally. Some studies have examined school or family factors in relation to gender differences in adolescent non-mental health outcomes or to mental health outcomes without reporting on gender differences (e.g., Resnick et al., 1997; Kuperminc et al., 2001; Crespo et al., 2013).

## Materials and Methods

### Participants and Procedure

A total of 10,138 adolescents from five larger Croatian cities and regional centers (Zagreb, Split, Osijek, Pula, and Varaždin) were recruited to complete a survey questionnaire for this study. In all cities except Zagreb, all schools were included with the intent to reach a quota of 25% of all high-school students in each locality. Given the large population size of Zagreb, the capital of Croatia, the sample was stratified according to three Croatian high school education programs: general preparatory grammar education, 3-year educational schools, and 4-year educational schools. Three- and 4-year programs prepare students for employment in specific professions after graduation, while preparatory grammar school is intended for students planning to attend university. Zagreb’s local education authority provided the total number of students enrolled in all three types of education programs and 15% of the total student number was calculated and then stratified by the three education programs. The number of schools per stratum was calculated and each school was chosen according to the school size, average school performance, school location, and gender composition of students in order to achieve the maximum representation of the Zagreb high school student population. The number of included students from each school was equal to the school size proportion in the total sample. A total of 77 schools were included in the sample.

Participants were aged between 14–19 years (*M* = 16.3, *SD* = 1.2). 52.5% of participants were female, and 26.5% were enrolled in a professional 3-year education program, 49.9% in a professional 4-year education program, and 23.6% in a general education (university preparation) program. The program distribution closely represented the national distribution of students enrolled in each program. Participants were also approximately equally distributed across first, second, and third years of programs, with a lower number of fourth-year students due to the presence of 3-year school programs in the sample. Socio-demographic characteristics of participants are presented in Table 1.

**TABLE 1:** Socio-demographic characteristics of participants by data collection site.

	**Zagreb**	**Split**	**Osijek**	**Pula**	**Varaždin**
*N* (%)	4430 (45.9)	1236 (12.8)	1675 (17.3)	704 (7.3)	1610 (16.7)
Age, *n* (%)^a^					
14	157 (3.5)	—	50 (3.0)	15 (2.1)	73 (4.5)
15	1342 (30.3)	255 (20.6)	469 (28.0)	216 (30.7)	450 (28.0)
16	1183 (26.7)	349 (28.2)	433 (25.9)	163 (23.2)	387 (24.0)
17	1079 (24.4)	340 (27.5)	427 (25.5)	163 (23.2)	407 (25.3)
18	619 (14.0)	247 (20.0)	280 (16.7)	138 (19.6)	268 (16.6)
19	39 (0.9)	45 (3.6)	14 (0.8)	9 (1.3)	25 (1.6)
Gender, *n* (%)					
Female	2318 (52.3)	669 (54.1)	901 (53.8)	427 (60.7)	762 (47.3)
Male	2112 (47.7)	567 (45.9)	774 (46.2)	277 (39.3)	848 (52.7)

*^*a*^13 (0.1%) participants were missing age information.*

Ethical approval for the study was obtained from the Ministry of Science and Education, National Agency for Education and Ethical Committee at the Faculty for Education and Rehabilitation Sciences, University of Zagreb. The study was also approved by the local education authorities (Offices for Education) within city or region level. The study team organized meetings with school headmasters and school counselors in each of five sites to obtaining their approval and partnership. Letters were sent in order to inform parents that the study was taking place. According to the Ethical Codex for Research with Children (Ajduković and Kolesarić, 2003), adolescents that are 14 years old can give their consent autonomously. Their written consent was obtained after the study objectives were explained and before the survey was taken. Participation was voluntary and fully confidential. The survey was administered during school hours, and filled individually by each participant. The average time required to complete the questionnaire was approximately 40 min, and the data collection process was conducted by study researchers and trained graduate and undergraduate students.

### Measures

All measures were either constructed in Croatian (Roviš and Bezinović, 2011) or translated from English and validated in previous research studies conducted in Croatia (e.g., project FamResPlan, funded by Croatian Science Foundation, grant number IP-2014-09-9515; Maurović et al., 2020).

#### School Factors

School connectedness was assessed with the 17-item School Bonding Scale (Roviš and Bezinović, 2011), which has two subscales measuring *attachment to teachers and school* (α = 0.89) and *commitment to schooling* (α = 0.89). Examples of the attachment items include, “I am satisfied with my teachers” and “Teachers treat us with care and respect.” Commitment items include, “I study regularly” and “I strive to be a better student.” All items were endorsed using a four-point rating scale, with response options ranging from “never” to “very often.”

#### Family Factors

The Family Satisfaction Scale (Olson and Gorall, 2006) questionnaire contains 10 items (α = 0.94) related to family life satisfaction. Examples of items include, “How satisfied are you with the degree of closeness with your family members?” and “How satisfied are you with your family’s ability to solve problems?” For each statement, participants endorsed a five-point Likert-type scale, from “very dissatisfied” to “very satisfied.”

The Family Communication Scale (Olson and Gorall, 2006) questionnaire contains 10 items (α = 0.93) on aspects of communication within the family (e.g., “Our family members are satisfied with how they communicate with each other” and “When angry, our family members seldom say negative things to each other”). For each statement, participants endorsed a five-point Likert-type scale, from “strongly disagree” to “completely agree.”

#### Mental Health Status

The Depression, Anxiety, and Stress Scale–21 Items (DASS-21) is a self-reported assessment with three scales designed to measure the emotional states of depression, anxiety, and stress (Lovibond and Lovibond, 1995). Each of the three DASS-21 scales contains seven items and a four-point rating scale ranging from 0 (did not apply to me at all) to 3 (applied to me very much or most of the time). The depression scale (α = 0.89) assesses dysphoria, hopelessness, devaluation of life, self-deprecation, lack of interest, and inertia. The anxiety scale (α = 0.85) assesses autonomic arousal, skeletal muscle effects, situational anxiety, and subjective experiences of anxious affect. The stress scale (α = 0.88) is sensitive to levels of chronic non-specific arousal. It assesses difficulty relaxing, nervous arousal, and being easily upset or agitated, irritable or over-reactive, and impatient.

### Statistical Analyses

Structural equation modeling (SEM; e.g., Bentler, 1980; Bollen, 1989; Kline, 2016) was used to investigate the relations between school and family factors and depression, anxiety, and stress outcomes. SEM differs from traditional observed data analysis (e.g., multiple linear regression) by including both observed and latent variables. Latent variables represent constructs of interest, such as family satisfaction or depression, and are estimated using information available from all measures (i.e., every item of scales used to assess predictors or outcomes). Further, SEM permits the separate specification and testing of measurement and structural models. The measurement model describes the relation of observed (measurement) variables with latent variables. In the case of a depression latent variable, for instance, the measurement model specifies which scale items are theorized or designed to assess depression. The structural model, by contrast, specifies the relations among latent variables (or a mixture of latent and observed variables) that are to be tested, such as whether family satisfaction is associated with depression. Finally, unlike observed linear modeling approaches, SEM does not assume that constructs of interest are measured without error.

**FIGURE 1:**
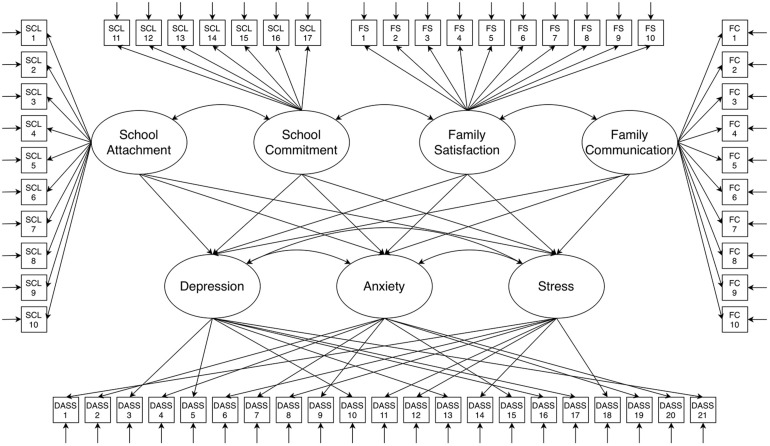
Structural equation model of the association of school attachment, school commitment, family communication, and family satisfaction with depression, anxiety, and stress. Oval figures are latent variables, estimated using observed scale variables represented by rectangular figures. Straight, open-ended arrows adjoining observed scale variables indicate residual error terms, which are estimated as latent variables. Curved arrows between latent variables denote correlations.

Because of these attributes, SEM offers numerous benefits over traditional observed data analysis. These include, foremost, the ability to examine the validity and properties (including error) of the measurement model, and to use all available data. Using information from all measures avoids the need to create scale summary scores, which can have problematic or poorly understood measurement and analytic properties, and allows missing data to be addressed during model estimation (when missing data are missing completely at random or missing at random). SEM also provides informative model fit and precision indices, and estimation approaches can be used that are robust to non-normality (Kline, 2016). Figure 1 presents the model used in the current study, in which the association of school attachment, school commitment, family communication, and family satisfaction latent variables with latent variables representing depression, anxiety, and stress is assessed. Rectangular figures indicate the observed scale variables used to estimate each latent variable. Curved lines between latent variables denote correlations estimated between those variables.

The procedure used to fit the above model was as follows. First, although scales used for predictors and outcomes had been previously validated (see section “Measures”), their measurement properties in the present sample were examined using confirmatory factor analysis. Once a satisfactory measurement model for each construct was established, a structural model was specified representing the hypothesized relations among latent variables. Absolute fit of confirmatory factor and full models was assessed using model root-mean-square error of approximation (RMSEA) and standardized root mean square residual (SRMR), as well as a test of close fit (the probability that RMSEA is below 0.05). Comparative fit was evaluated using the comparative fit index (CFI), Tucker–Lewis index (TLI), and a test of the difference in chi-square statistics between alternate models (Δχ^2^). Finally, comparative and parsimonious fit was assessed using change in model Bayesian information criterion (BIC) value. Because the chi-square test of overall model fit is known to be overpowered in large samples (e.g., Shi et al., 2019), this fit statistic is not reported.

All models were fitted with M*plus* version 8.4 (Muthén and Muthén, 2019) using maximum likelihood estimation. Robust (sandwich) standard errors were estimated. Models were specified *a priori* as multigroup models with relations between predictors and outcomes allowed to differ for female and male adolescents.^3^ 4.8% of adolescents (*n* = 483) did not provide information on gender or provided no responses to questionnaire items, and were not included in the analytic sample (*N* = 9,655). Any remaining missing response data (2.4%) were addressed using a full information maximum likelihood approach during model estimation. Standardization of observed and latent variables was employed, and statistical significance of associations was evaluated at a significance level of α = 0.05. Prior to confirmatory factor and full latent variable model fitting, an unconditional multilevel model was fitted to assess whether variation in outcomes occurred at city or school levels. No substantial variation was identified at either level (all intraclass correlation coefficients < 0.04), and consequently single level models were fitted for all subsequent stages of modeling.

## Results

Descriptive statistics of observed predictor and outcome scale scores for male (*n* = 5,074) and female (*n* = 4566) adolescents are presented in Table 2. Compared with male adolescents, female adolescents on average reported significantly higher school commitment, depression, anxiety, and stress scale scores. Male adolescents reported significantly higher scores on family communication and satisfaction scales on average. There was no difference between female and male adolescents in mean score on the school attachment scale.

**TABLE 2:** Descriptive statistics of observed predictor and outcome scale scores.

		**Female (*n* = 5,074)**	**Male (*n* = 4,566)**	
	**Scale range**	**Mean (*SD*)**	**Mean (*SD*)**	***p*-value**
School attachment	0–30	16.2 (5.7)	16.2 (6.4)	0.974
School commitment	0–21	12.8 (4.9)	11.4 (5.2)	**<0.001**
Family communication	0–50	38.1 (9.7)	39.7 (9.3)	**<0.001**
Family satisfaction	0–50	37.6 (9.4)	39.7 (8.5)	**<0.001**
Depression	0–21	7.0 (5.7)	5.1 (4.8)	**<0.001**
Anxiety	0–21	6.9 (5.4)	4.8 (4.5)	**<0.001**
Stress	0–21	8.8 (5.7)	6.1 (4.9)	**<0.001**

*Boldface indicates statistically significant differences between female and male students using a Welch’s *t*-test for independent samples and a significance level of α = 0.05.*

The initial confirmatory factor model was adequately fitting; substantially improved fit was achieved by permitting covariances and residual covariances among several scale variables to be freely estimated (RMSEA = 0.04; probability RMSEA < 0.05 = 1.00; SRMR = 0.03; CFI = 0.95; TLI = 0.95; (Δχ^2^) *p*-value < 0.001; reduction in BIC value = 11032.20).

The final full model was well fitting with respect to absolute fit indices (RMSEA = 0.04, probability RMSEA < 0.05 = 1.00, SRMR = 0.03) and comparative fit indices (CFI = 0.95, TLI = 0.95). As a final step in model fitting, a sensitivity analysis was carried out to assess whether freely estimating some covariances and residual covariances altered final model interpretation. Final model results and interpretation did not substantively differ from a full latent variable model without the modifications, and as a result they were retained.^4^

Results of the final model assessing the relation of school and family factors with student depression, anxiety, and stress are presented in Table 3. The first school factor examined, school attachment, was found to be significantly associated with depression, anxiety, and stress for female adolescents and with depression and stress for male adolescents. For female adolescents, each standard deviation (*SD*) increase in school attachment was associated with a 0.09 *SD* decrease in depression, 95% CI [−0.13, −0.06]; with a 0.06 *SD* reduction in anxiety, 95% CI [−0.10, −0.02]; and with a 0.11 *SD* decrease in stress, 95% CI [−0.15, −0.08]. For male adolescents, each *SD* increase in attachment was associated with a 0.06 *SD* decrease in depression, 95% CI [−0.10, −0.02], and with a 0.09 *SD* decrease in stress, 95% CI [−0.13, −0.05].

**TABLE 3:** Results of structural equation model examining the association of school attachment, school commitment, family communication, and family satisfaction with female and male student depression, anxiety, and stress.

	**Female (*n* = 5,077)**			**Male (*n* = 4,578)**		
	**β**	***p-*value**	**95% CI**	**β**	***p-*value**	**95% CI**
*School*						
Attachment						
Depression	−0.09	**<0.001**	[−0.13, −0.06]	−0.06	**0.007**	[−0.10, −0.02]
Anxiety	−0.06	**<0.001**	[−0.10, −0.02]	−0.04	0.061	[−0.08, 0.02]
Stress	−0.11	**<0.001**	[−0.15, −0.08]	−0.09	**<0.001**	[−0.13, −0.05]
Commitment						
Depression	−0.11	**<0.001**	[−0.15, −0.08]	−0.02	0.409	[−0.06, 0.02]
Anxiety	−0.05	**0.016**	[−0.08, −0.01]	0.06	**0.004**	[0.02, 0.10]
Stress	−0.02	0.386	[−0.05, 0.02]	0.06	**0.002**	[0.02, 0.10]
*Family*						
Communication						
Depression	−0.06	0.184	[−0.14, 0.03]	−0.22	**<0.001**	[−0.28, −0.17]
Anxiety	−0.03	0.555	[−0.12, 0.06]	−0.26	**<0.001**	[−0.32, −0.20]
Stress	−0.01	0.880	[−0.10, 0.08]	−0.24	**<0.001**	[−0.30, −0.18]
Satisfaction						
Depression	−0.33	**<0.001**	[−0.42, −0.25]	−0.16	**<0.001**	[−0.21, −0.10]
Anxiety	−0.28	**<0.001**	[−0.37, −0.19]	−0.04	0.146	[−0.10, 0.02]
Stress	−0.34	**<0.001**	[−0.43, −0.26]	−0.10	**<0.001**	[−0.16, −0.05]
*Model fit*						
RMSEA, *P*(<0.05)	0.04 (1.00)					
SRMR	0.03					
CFI	0.95					
TLI	0.95					

*Boldface indicates statistically significant results using a significance level of α = 0.05. β, standardized model coefficient; CI, 95% confidence interval; RMSEA, *P*(<0.05), root-mean-square error of approximation, probability that RMSEA is less than 0.05 (test of close fit); SRMR, standardized root-mean-square residual; CFI, comparative fit index; TLI, Tucker–Lewis index.*

The second school factor, school commitment, was significantly associated with depression and anxiety for female adolescents. Each *SD* increase in commitment was associated with a 0.11 *SD* decrease in depression, 95% CI [−0.15, −0.08], and with a 0.05 *SD* decrease in anxiety, 95% CI [−0.08, −0.01]. For male adolescents, school commitment was associated only with anxiety and stress; conversely to females, a one *SD* increase in commitment was associated with a 0.06 *SD* increase in anxiety, 95% CI [0.02, 0.10], and with a 0.06 *SD* increase in stress, 95% CI [0.02, 0.10].

The family communication factor was significantly associated with depression, anxiety, and stress only for male adolescents. Each *SD* increase in family communication was associated with a 0.22 *SD* decrease in depression, 95% CI [−0.28, −0.17]; with a 0.26 *SD* reduction in anxiety, 95% CI [−0.32, −0.20]; and with a 0.24 *SD* decrease in stress, 95% CI [−0.30, −0.18]. The second family factor, family satisfaction, was significantly associated with all latent outcome variables for female adolescents. Each *SD* increase in satisfaction was associated with a 0.33 *SD* decrease in depression, 95% CI [−0.42, −0.25]; with a 0.28 *SD* reduction in anxiety, 95% CI [−0.37, −0.19]; and with a 0.34 *SD* decrease in stress, 95% CI [−0.43, −0.26]. For male adolescents, family satisfaction was significantly associated with depression and stress, with each *SD* increase in family satisfaction associated with a 0.16 *SD* decrease in depression, 95% CI [−0.21, −0.10], and with a 0.10 *SD* decrease in stress, 95% CI [−0.16, −0.05].

Finally, correlations among latent variables were examined. Depression, anxiety, and stress were highly correlated for both female and male adolescents (range = 0.83–0.90, all *p*-values < 0.001). School attachment and commitment were moderately correlated for both female and male adolescents (range = 0.49–0.56, all *p*-values < 0.001), while family satisfaction and communication were highly correlated for both female and male adolescents (range = 0.81–0.92, all *p*-values < 0.001). Family satisfaction and communication were not highly correlated with school attachment and commitment for female nor male adolescents (range = 0.23–0.28, all *p*-values < 0.001). Table 4 presents a correlation matrix and factor loading information for all latent variables.

**TABLE 4:** Covariance matrix and factor loadings of latent outcome and predictor variables.

	**Depress.**	**Anxiety**	**Stress**	**School bonding**	**School commit.**	**Family comm.**	**Family satis.**
Depression	1.00						
Anxiety	0.88	1.00					
Stress	0.90	0.91	1.00				
School attachment	−0.16	−0.08	−0.14	1.00			
School commitment	−0.15	−0.05	−0.08	0.56	1.00		
Family communication	−0.37	−0.29	−0.33	0.26	0.28	1.00	
Family satisfaction	−0.35	−0.24	−0.30	0.23	0.25	0.81	1.00
*Factor loadings*							
Range	0.50–0.72	0.44–0.73	0.54–0.74	0.45–0.66	0.56–0.77	0.68–0.99	0.72–1.02
Mean	0.65	0.62	0.66	0.56	0.66	0.85	0.87

## Discussion

Internalizing symptoms during adolescence are increasing, especially among female adolescents, who are more than twice as likely as male adolescents to experience symptoms of depression or anxiety (Costello et al., 2003; Kessler et al., 2007; Merikangas et al., 2010; Bor et al., 2014; Dooley et al., 2015). The present study examined the associations of school attachment, school commitment, family communication, and family satisfaction with depression, anxiety, and stress symptoms in a large, population-representative sample of Croatian youth. Further, we examined how these relations differed for male and female adolescents to enable a deeper understanding of the role of school and family factors in the quality of Croatian youth mental health. Findings show that school and family factors are related to depression, anxiety, and stress symptoms in distinct ways for female and male adolescents.

### School Attachment and School Commitment

Findings for school attachment and its relation with depression, anxiety, and stress symptoms align with previous research (Resnick et al., 1997; Anderman, 2002; Whitlock, 2003; Shochet et al., 2006; Costello et al., 2008; Loukas et al., 2009; Kidger et al., 2012; Langille et al., 2012; Dooley et al., 2015). In the present sample, female and male adolescents endorsed similar levels of attachment to teachers and school. Female adolescents with higher school attachment were found to have reduced depression, anxiety and stress, and male adolescents were found to have reduced depression and stress. These findings add to other similar findings suggesting school attachment can serve as a protective factor. For example, Joyce and Early (2014) found that better school connection and student-teacher relationship quality were associated with fewer depressive symptoms. Similarly, Foster et al. (2017) found that youth who report more connectedness to school reported fewer depressive symptoms, less suicidal ideation, and lower levels of social anxiety. Indeed, school connectedness may be one of the most important factors in PYD, in that it buffers youth mental health against other adversities (Pate et al., 2017).

In line with our hypothesis, associations between school attachment and commitment and mental health indicators were stronger for adolescent females. It is conceivable that this finding may be, in part, due to different levels of emotional connection to school and peers among adolescent females compared with adolescent males. For example, Shochet et al. (2006) found that concerns about acceptance and connections to teachers and peers are more powerful predictors of anxiety in female students than male students. Additionally, relations of both school attachment and school commitment with depression, anxiety, and stress may be bidirectional for both genders. Experiencing emotional distress as an adolescent may affect sense of connectedness as well as commitment to obligations. It is also plausible that youth who do not experience internalizing symptoms have more positive relationships with peers and find it easier to connect with their school environment. Lester et al. (2013), however, found that school connectedness is a more powerful predictor for mental health outcomes than the reverse.

Finally, although our results indicate a stronger relation among both school factors and mental health outcomes for female compared with male adolescents, for males school attachment was still an important predictor of the mental health outcomes considered in this study. In our study, school commitment was significantly associated with decreased depression and anxiety for female adolescents. Bor et al. (2014) found that female adolescents are more affected by pressure to have high academic performance, and it is plausible greater commitment to school may give female students a greater sense of achievement and purpose in the school environment. This state may positively impact their mental health. For male adolescents, by contrast, an increase in school commitment was associated with an increase in anxiety and stress. This finding aligns with an earlier study showing that male students experience significantly higher levels of chronic academic stress compared to female students (Shih et al., 2006), and it may be the case that engagement in academic activities leads to greater stress and anxiety for male students than for female students. Nevertheless, this finding should be replicated and further investigated in other samples.

### Family Communication and Family Satisfaction

Evidence on the associations between family functioning factors and internalizing problems in adolescence is relatively scarce, in particular for gender differences in these relations. Our findings go some way to addressing these gaps. First, in general larger magnitude associations with depression, anxiety, and stress were found for family factors compared to school factors. Further, male and female adolescents showed different associations between family communication and family satisfaction and their mental health. Assessing the significance and magnitude of the associations, family satisfaction appears to be more important for the mental health of adolescent females, while family communication may have greater importance for the mental health of adolescent males. Interestingly, descriptive findings suggest that female adolescents are more critical of family functioning, which may indicate they have elevated standards for family life compared to male adolescents.

Results showing an absence of significant association between family communication and mental health outcomes for female adolescents should be further investigated. It may be that because of socialization that instructs male adolescents to limit display of emotions, when families do communicate about emotional health, it has an especially large impact on male adolescents’ mental health. In contrast, emotionality may be generally more permissible and normed for female adolescents, and therefore greater family communication may not make meaningful differences in mental health outcomes (i.e., such topics may already be more freely discussed). Exploring these dynamics could help to clarify the existing mixed research findings. For example, some studies have found that adolescent internalizing disorders negatively impact family functioning (Branje et al., 2010; Elgar et al., 2013; Mastrotheodoros et al., 2019). In contrast, other studies have found poor family communication to be associated with higher adolescent anxiety and depression (Bögels and Brechman-Toussaint, 2006; Smokowski et al., 2015; Withers et al., 2016) and lower self-esteem (Smokowski et al., 2015). Finally, Mastrotheodoros et al. (2019) did not find any associations of family functioning and adolescent internalized problems on the family level.

Findings indicate family satisfaction is associated with depression and stress for both male and female adolescents, with a larger association for female adolescents. These results align with those of Chen and Harris (2019). In addition, family satisfaction is not significantly associated with anxiety for male adolescents while it is strongly associated with anxiety for female adolescents in our sample. It may be that female adolescents are more aware of family dysfunction and interpersonal stressors than male adolescents. Telzer and Fuligni (2013) found that female adolescents who experience positive family interactions report fewer internalizing symptoms. Despite some differences by gender, our findings along with those of prior studies underline the importance of family relationships for the mental health of both female and male adolescents.

### Recommendations for Policy and Practice

To approach youth mental health comprehensively, preventive interventions should focus on improving the relational context of schools and families, investing in capacities of teachers to relate with students more meaningfully and providing tools for better family functioning. Schools represent one of the most important community settings where the mental health of young people can be promoted. For example, Waters et al. (2009) identified four school-associated factors that contribute most to school connectedness: (1) organizational structure (e.g., smaller schools); (2) functional aspects of schools (e.g., clearly defined disciplinary expectations); (3) the built environment of the school (e.g., well-maintained facilities); and (4) interpersonal support (e.g., positive relationships among students and between staff and students). Interventions focused on enhancing the quality of these factors could have a substantial beneficial effect on students’ mental health. Reviews of the literature suggest that mental health promotion programs in schools, especially those adopting an approach focusing on psychosocial competence rather than specific behavioral problems, produce long-term benefits for young people, including improved emotional and social functioning and positive health behavior as well as improvement in school climate, teacher–student relationships, and teacher stress reduction (Durlak et al., 2011; Barry et al., 2013).

The family has a vital and unique role in providing a supportive environment for PYD, buffering the negative impacts of various stressors on youth mental health (Kaminski et al., 2008; Cartwright-Hatton et al., 2011; Barry et al., 2013; Lewis et al., 2013; Chu et al., 2015). Yet, parents of adolescents often need tools that improve their responses to conflict and that help them to focus on positive events in daily life. Quality family-based programs should focus on strengthening parenting and family relational skills, supporting parent/child attachment and positive interactions, creating warm and supportive family environments, communicating effectively, monitoring appropriately, and displaying effective discipline skills across adolescence (Jacka et al., 2013; Kumpfer, 2014).

## Limitations and Conclusion

This study has several limitations. First, as a cross-sectional design, the causal relations among school and family factors and depression, anxiety, and stress could not be examined. Future longitudinal studies are needed to examine the directionality of these relations. Future studies should also include measures of parental mental health, parent–child relationship quality, family socioeconomic status, and context (e.g., urban vs. rural). Regarding school factors, some important constructs were not incorporated into this study, including academic motivation, school climate, or parents’ involvement in school. Additionally, only self-reported measures were used, and there may have also been underreporting of mental health outcomes by male adolescents due to concerns about adherence to gender norms (Kerr and Kerr, 2001).

Despite the above limitations, findings of this study emphasize the importance of school and family contexts to adolescent mental health, including levels of depression, anxiety, and stress. Observed differences in multi-group models also suggest that strategies for mental health promotion and prevention planning may need to be tailored to female and male adolescents. More generally, evidence-based knowledge translation strategies should be developed to ensure that all stakeholders recognize the importance of preventing mental health disorders among adolescents and young adults, with appropriate resources directed to this objective (Jacka et al., 2013). To this end, the availability of evidence-based prevention programs in family, school, and community settings is crucial for comprehensive and effective youth mental health promotion strategies. Programs effective in promoting PYD involve key features known as the Big Three (Tirrell et al., 2020) or core competencies (Maslow and Chung, 2013): (1) positive and sustained adult–youth relationships, (2) life-skill-building activities, and (3) opportunities for youth contribution and leadership. These principles should be enacted in all environments, especially in schools and families.

## Data Availability Statement

The raw data supporting the conclusions of this article will be made available by the authors, without undue reservation.

## Ethics Statement

Ethical approval for the study was obtained from the Ministry of Science and Education, National Agency for Education, and Ethical Committee at the Faculty for Education and Rehabilitation Sciences, University of Zagreb. Written informed consent for participation was not provided by the participants’ legal guardians/next of kin because: According to the Ethical Codex for Research with Children (Ajduković and Kolesarić, 2003), adolescents that are 14 years old can give their consent autonomously. Their written consent was obtained after the study objectives were explained and before the survey was taken.

## Author Contributions

MN, MF, JM, and VK designed the study and participated in data collection in the city of Zagreb. MN coordinated the data collection on other sites and supervised the project. NP analyzed the data and prepared analysis and result sections. All authors contributed to drafting and revising the manuscript.

## Conflict of Interest

The authors declare that the research was conducted in the absence of any commercial or financial relationships that could be construed as a potential conflict of interest.

## Figures and Tables

**Appendix Table 1:** Results of ungrouped structural equation model on the association of school attachment, school commitment, family communication, and family satisfaction with female and male student depression, anxiety, and stress.

	**β**	***p-*value**	**95% CI**
*School*			
Attachment			
Depression	−0.09	**<0.001**	[−0.11, −0.06]
Anxiety	−0.07	**<0.001**	[−0.09, −0.04]
Stress	−0.12	**<0.001**	[−0.15, −0.09]
Commitment			
Depression	−0.02	0.061	[−0.05, 0.00]
Anxiety	0.06	**<0.001**	[0.03, 0.08]
Stress	0.08	**<0.001**	[0.05, 0.10]
*Family*			
Communication			
Depression	−0.18	**<0.001**	[−0.22, −0.13]
Anxiety	−0.18	**<0.001**	[−0.23, −0.13]
Stress	−0.17	**<0.001**	[−0.22, −0.12]
Satisfaction			
Depression	−0.24	**<0.001**	[−0.28, −0.19]
Anxiety	−0.15	**<0.001**	[−0.20, −0.10]
Stress	−0.21	**<0.001**	[−0.25, −0.16]
*Model fit*			
RMSEA, *P*(<0.05)	0.03 (1.00)		
SRMR	0.03		
CFI	0.95		
TLI	0.95		

*Boldface indicates statistically significant results using a significance level of α = 0.05. β, standardized model coefficient; CI, 95% confidence interval; RMSEA, *P*(<0.05), root-mean-square error of approximation, probability that RMSEA is less than 0.05 (test of close fit); SRMR, standardized root-mean-square residual; CFI, comparative fit index; TLI, Tucker–Lewis index.*

## Footnotes

1 In this study, we use the term *gender*, rather than *sex*, to recognize that the relation of male and female identities with associations between school and family factors and adolescent mental health likely reflects a complex interplay of genetic, environmental, and sociocultural influences.

2 https://ec.europa.eu/eurostat/statistics-explained/index.php/Unemployment_statistics

3 Although not discussed here, results of an ungrouped model are presented in the Appendix.

4 The coefficient for the association of school attachment and anxiety among male adolescents was the same magnitude in both models (−0.04) but was non-significant in the final model (*p* = 0.033 vs. 0.061).
